# HAART induced inflammation, toxicity and its determinants among HIV positive children in Addis Ababa, Ethiopia

**DOI:** 10.1016/j.heliyon.2023.e15779

**Published:** 2023-04-28

**Authors:** Yimam Getaneh, Tadesse Lejissa, Tigist Getahun, Siti qamariyah khairunisa, Dominicus Husada, Kuntaman Kuntaman, Maria Inge Lusida

**Affiliations:** a Doctoral Program, Faculty of Medicine, Universitas Airlangga, Indonesia; b Ethiopian Public Health Institute, Indonesia; c Research Center on Global Emerging and Re-emerging Infectious Diseases, Institute of Tropical Disease, Universitas Airlangga, Surabaya, 60115, Indonesia

**Keywords:** Inflammation, Liver toxicity, Renal toxicity, Hematologic toxicity

## Abstract

**Background:**

Highly Active Antiretroviral therapy (HAART) plays significant role in reduction of mortality among children infected with HIV. Despite the inevitable impact of HAART on inflammation and toxicity, there is limited evidence on its impact among children in Ethiopia. Moreover, evidence on contributing factors to toxicity has been poorly described. Hence, we evaluated HAART induced inflammation and toxicity among children taking HAART in Ethiopia.

**Method:**

This cross-sectional study was conducted among children (<15 years old) taking HAART in Ethiopia. Stored plasma samples and secondary data from a previous study on HIV-1 treatment failure were used for this analysis. By 2018, a total of 554 children were recruited from randomly selected 43 health facilities in Ethiopia. The different levels of liver (SGPT), renal (Creatinine) and hematologic toxicity (Hemoglobin) toxicity were assessed using established cut-off value. Inflammatory biomarkers (CRP and vitamin-D) were also determined. Laboratory tests were done at the national clinical chemistry laboratory. Clinical and baseline laboratory data were retrieved from the participant's medical record. Questionnaire was also administered to study guardians to assess individual factors to inflammation and toxicity. Descriptive statistics was used to summarize the characteristics of the study participants. Multivariable analysis was conducted and considered significant at P < 0.05.

**Result:**

Overall 363 (65.6%) and 199 (36%) of children taking HAART in Ethiopia developed some level of inflammation and vitamin-D in-sufficiency, respectively. A quarter of the children 140 (25.3%) were at Grade-4 liver toxicity while renal toxicity were 16 (2.9%). A third 275 (29.6%) of the children also developed anemia. Children who were on TDF+3 TC + EFV, those who were not virally suppressed and children with liver toxicity were at 17.84 (95%CI = 16.98, 18.82), 2.2 (95%CI = 1.67, 2.88) and 1.20 (95%CI = 1.14, 1.93) times risk of inflammation, respectively. Children on TDF+3 TC + EFV, those with CD4 count of <200 cells/mm^3^ and with renal toxicity were at 4.10 (95%CI = 1.64, 6.89), 2.16(95%CI = 1.31, 4.26) and 5.94 (95%CI = 1.18, 29.89) times risk of vitamin-D in-sufficiency, respectively. Predictors of liver toxicity were history of HAART substitution (AOR = 4.66; 95%CI = 1.84, 6.04) and being bedridden (AOR = 3.56; 95%CI = 2.01, 4.71). Children from HIV positive mother were at 4.07 (95%CI = 2.30, 6.09) times risk of renal toxicity while the different type of HAARTs had different level of risk for renal toxicity AZT+3 TC + EFV (AOR = 17.63; 95%CI = 18.25, 27.54); AZT+3 TC + NVP (AOR = 22.48; 95%CI = 13.93, 29.31); d4t+3 TC + EFV (AOR = 4.34; 95%CI = 2.51, 6.80) and d4t+3 TC + NVP (AOR = 18.91; 95%CI = 4.87, 27.74) compared to those who were on TDF+3 TC + NVP. Similarly, children who were on AZT+3 TC + EFV were at 4.92 (95%CI = 1.86, 12.70) times risk of anemia compared to those who were on TDF+ 3 TC + EFZ.

**Conclusion:**

The high level of HAART induced inflammation and liver toxicity among children calls for the program to consider safer regimens for pediatric patients. Moreover, the high proportion of vitamin-D in-sufficiency requires program level supplement. The impact of TDF+3 TC + EFV on inflammation and vitamin-D deficiency calls for the program to revise this regimen.

## 1 Background

Highly Active Antiretroviral Therapy (HAART) has changed human immunodeficiency virus (HIV) infection from a deadly illness to a chronically managed one [1]. With the increased access to HAART, clinicians should be able to recognize the common toxicities associated with HAART so that to improve treatment outcome of people living with HIV (PLHIV) [2]. In spite of the benefits of HAART, adverse effects, of which toxicity is a common finding which can lead to discontinuation, switch and non-adherence to therapy [3,4]. Data on the impact of antiretroviral therapy and specifically on individual antiretroviral agents remain conflicting [1,[3], [4], [5], [6], [7]].

A protective and contrary worsening effect has been reported for NVP and protease inhibitors [8]. Differences in study design and methodologies may account for discrepancies. Overall, it is well accepted that HAART is beneficial and protects from disease progression in HIV infected subjects with increased survival of HIV-infected persons due to improved combination antiretroviral therapy and viral suppression, complications in this population have increased as well [9]. These abnormalities could predispose the children to adverse drug reactions. In the HIV-infected population this may be further compounded by cumulative drug exposure, as diagnosis remains more prevalent in younger populations, and earlier initiation of antiretroviral therapy is currently the rule [10]. This would commit HIV patients to life-long antiretroviral therapy, and therefore to more opportunities for developing toxicity.

Understanding of HIV treatment increases with extensive experience, and modern antiretroviral agents with improved safety profiles continue to develop as a result it is assumed the incidence of HAART-related toxicity decrease over time [11]. However, evidence in this field posed and lack of uniform definition and complexity of both patients and treatment regimens remains a challenge in the field. Moreover, HAART regimens are not yet validated for children while this group of population has a different and complex physiology. As a result, children taking HAART might be significantly affected by the different toxicities including hepatotoxicity, nephrotoxicity, hematologic toxicity which could initially be explained by HAART induced inflammation. In this regard, there limited and controversial evidence on the impact of HAART on toxicities and inflammation which could be more worsening among children.

In Ethiopia there were picket studies conducted on the effect of HAART on toxicity among adult population and yet the findings were not conclusive [3,[6], [7], [8]]. However, children were not part of these studies while they are presumed to be at high risk. Hence, our current study was aimed at evaluating inflammation, toxicities and their determinants among children taking HAART in Ethiopia.

## 2 Method

A cross-sectional study was conducted from November 2021–June 2022. Stored plasma samples and secondary data from the nationally representative research project titled: “HIV-1 treatment failure and acquired drug resistance among first line antiretroviral experienced patients in Ethiopia” was used for this study. Study participants were recruited from randomly selected 43 health facilities in Ethiopia (18 hospitals and 25 health centers). A total of 554 children (<15 years old) were included in this study. Clinical and laboratory data were captured from the participant's medical record. Moreover, structured questionnaire was administered to mothers or female guardians of the study participants to assess determinants of inflammation and toxicity among children taking HAART. The source population for this study was children who are on HAART in Ethiopia. Children attending the selected 43 health facilities who were taking HAART for at least 6 months were eligible for this study.

### 2.1 Sampling and sample size determination

Systematic random sampling was used to select study participants in this study to determine proportion of population with different level of inflammation and toxicity among children on HAART in Ethiopia. The required sample size for this study was by assuming the worst acceptable 50% population proportion with 95% confidence interval and 5% margin of error. The sample size was calculated with using open epi formula available at:

http://www.openepi.com/SampleSize/SSCohort.htm.

Accordingly, the minimum sample was calculated as 370. Considering a design effect of 1.5, the estimated sample size was 555.

## 3 Laboratory testing

Toxicity biomarker tests for renal toxicity (creatinine) and hepatotoxicity (baseline SGPT) were done at the health facilities where the children attending for HAART. As follow-up, the most recent creatinine and SGPT test was done at the national Clinical Chemistry laboratory. Moreover, CRP and Vitamin-D was done by using Elecsys 2010 Clinical Chemistry Analyzer (Roche).

According to the national guideline for children, during HAART initiation, it is a requirement to see hematological profiles. Hence, we retrieved hemoglobin results from children across their entire cohort from their medical record at the health facility. Moreover, the most recent hemoglobin test was done at the national hematology laboratory using CELDYN hematology analyzer.

CD4 count of the children taking HAART was archived from the medical record of the participant while the latest CD4 count was done at the national Hematology laboratory using BD FACS count machine. HIV-1 viral load testing was done at regional laboratories in Ethiopia and 10% quality check was done at EPHI, by using Abbott m2000rt PCR.

### 3.1 Dependent and independent variables

The dependent variables were hepatotoxicity as measured from Grade-0 to 4 as per the definition of the Global AIDS Clinical Trial Group [12,5,13]. Nephrotoxicity was also measured by the cutoff value of the level of Creatinine using the predefined reference range [[13], [14], [15]]. Hematologic toxicity was defined using the hemoglobin cut off value established locally [16] and inflammation was defined by considering CRP and Vitamin-D as defined by Refs. [[17], [18], [19], [20]] (Table-1).

**Table-1:** Reference ranges for inflammation and toxicity among children taking HAART in Ethiopia.

Toxicity	Stage	Range
Liver toxicity (Jones and Núñez, 2012)	Grade-0	<1.25 X baseline AT
	Grade-1	1.25–2.5 X baseline AT
	Grade-2	2.6–3.5 X baseline AT
	Grade-3	3.6–5 X baseline AT
	Grade-4	>5 X baseline AT
Kidney (CKD) (Canal et al., 2008)	Stage-1	Creatinine<1.30 ml/dl
	Stage-2	Creatinine≥1.30 ml/dl
Hematologic (American College of Clinical Pharmacy, 2018)	Normal	Hgb≥11 mg/dl
	Anemic	Hgb<11 mg/dl
Vitamin-D (Canal et al., 2008)	Normal	≥20 ng/dl
	Vitamin D deficiency	<20 ng/dl
CRP (B, 2013)	Normal	0.0–8.0 mg/L
	High (Significant inflammation as a result of infection)	≥8.0 mg/L

Independent variables were socio-demographic characteristics (Age, residency, family status, sex, socio-economic and educational status) and clinical factors (type of HAART, duration on HAART, history of HAART substitution, history of OI, CD4 count, WHO clinical stage and medication adherence).

Functional status was categorized into two groups: normal activity and bedridden (>50% of the day during the past month) [28] as reported by the ART site physician. The WHO clinical stage was categorized into four groups (stage I, stage II, stage III, and stage IV) [29].

### 3.2 Statistical analysis

Descriptive analysis was done to evaluate the level of toxicities and inflammation. Factors associated with toxicity and inflammation was evaluated by comparing variables among children with toxicity and not, using the chi-square test for categorical data, and using student-t test for continuous variables. The model were then built by dropping the most insignificant factor one at a time with factors whose P < 0.05 were taken to be the factors that were independently associated with toxicity and inflammation. Bivariate analysis was done using 95% confidence intervals to compare factors in patients with toxicity to those without toxicity. Multivariable logistic regression analysis was done to characterize independent predictors of inflammation and toxicity. Briefly, a crude odds ratio (CoR) was calculated and a significant level of P < 0.2 were considered as candidate variable for adjusted odds ratio (AOR). All the analysis will be done using STATA software v16.0.

### 3.3 Ethical consideration

Ethical approval was obtained from the Ethiopian Public Health Institute Scientific and Ethical Review Office (SERO) with approval number; EPHI-IRB-191-2019. Confidentiality was respected during abstraction of data by the use of specific identification code for each enrolled participant. Eligible study participants were identified by trained and experienced data collectors and supervisor at facility level. Informed consent was obtained from the legal guardians of all the participants used in this study. For emancipated minor children, we also used ascent in addition to the consent from the legal guardian. All methods were performed in accordance with the relevant guidelines and regulations including the national IRB regulations, the journal guidelines and data protection protocol. Moreover, all the procedure was conducted according to the Helsinki declaration.

## 4 Result

### 4.1 Characteristics of the study participants

A total of 554 children, 275 (50.4%) males and 279 (49.6%) females were included in the study. Majority of the children were on AZT+3 TC + NVP 198 (35.7%) or d4t+3 TC + NVP 169 (30.5%). In total 344 (62.1%) of the study participants were virally suppressed. About half of the study participants were at WHO clinical stage-II 281 (50.7%) followed by WHO stage-I, 211 (37.1%). Overall, 405 (73%) of the study participants had good adherence while 413 (74.5%) were ambulatory. Majority of the children were attending for HAART from 71 to 100 months 385 (69.5%) (Table-2).

**Table-2:** Individual characteristics of Children taking HAART in Ethiopia (2018–2019).

Variables		Frequency	Percent
Gender	Female	275	49.6
	Male	279	50.4
Education status	No formal education	43	7.8
	Primary school education	444	80.1
	secondary/high school education	67	12.1
Family status	Yes	429	77.4
	No	19	3.4
	Don't Know	106	19.1
Type of HAART		35	6.3
	ABC,3 TC, EFV	9	1.6
	ABC,3 TC, LPV/r	1	0.2
	ABC,3 TC, NVP	1	0.2
	AZT,3 TC, EFV	77	13.9
	AZT,3 TC, LPV/r	2	0.4
	AZT,3 TC, NVP	198	35.7
	d4t,3 TC, EFV	25	4.5
	d4t,3 TC, NVP	169	30.5
	TDF,3 TC, EFV	35	6.3
	TDF,3 TC, NVP	2	0.4
HAART substitution	No	288	52.0
	Yes	266	48.0
Viral Suppression	Suppressed	344	62.1
	Not suppressed	210	37.9
Residency	Urban	497	89.7
	Rural	57	10.3
WHO clinical stage	I	211	38.1
	II	281	50.7
	III	51	9.2
	IV	11	2.0
Adherence	Good	405	73.1
	Fair	119	21.5
	Poor	30	5.4
Functional status	Ambulatory	413	74.5
	Bedridden	141	25.5
Age (Year)	≤1	1	0.2
	2–6	122	22.0
	7–11	259	46.8
	12–15	172	31.0
Duration on HAART (month)	≤10	1	0.2
	11–40	4	0.7
	41–70	99	17.9
	71–100	385	69.5
	101–131	61	11.0
	>132	4	0.7
	Total	554	100.0

Key: All characteristics were as of time of study.

### 4.2 Magnitude of inflammation and toxicity among children taking HAART in Ethiopia

**Figure-1:**
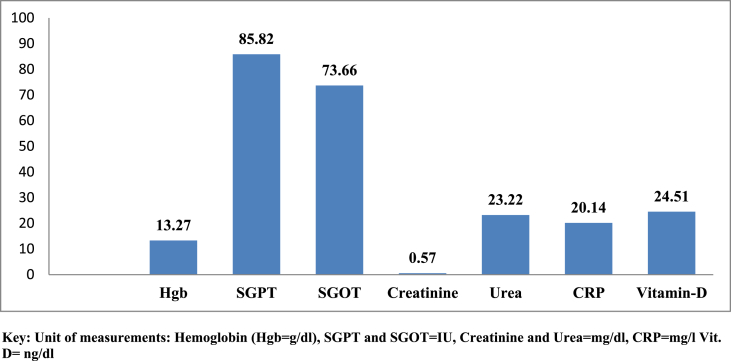
The mean levels of toxicities among children taking HAART in Ethiopia (2018–2019). Key: Unit of measurements: Hemoglobin (Hgb=g/dl), SGPT and SGOT=IU, Creatinine and Urea=mg/dl, CRP=mg/l Vit. D= ng/dl

The mean CD4 count, Hemoglobin, SGPT, SGOT, Creatinine, Urea, hsCRP and Vitamin-D among children taking HAART in Ethiopia was 1070.61 cells/mm^3^ (95%CI = 1014.08, 1127.14), 13.7 mg/dl (95%CI = 12.86, 13.69), 85.82 IU (95%CI = 82.0, 89.63), 73.66 IU (95%CI = 70.07, 77.24), 0.57 mg/dl (95%CI = 0.53,0.61), 23.22 mg/dl (95%CI = 22.53,23.91), 20.14 mg/dl (95%CI = 18.83, 21.44) and 24.51 mg/dl (95%CI = 23.73,25.30) respectively (Figure-1).

**Figure-2:**
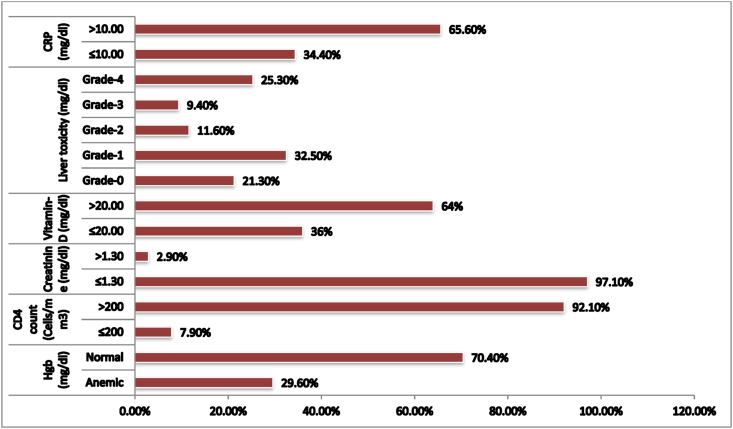
Magnitude of inflammation and toxicity among children taking HAART in Ethiopia (2018–2019).

Inflammation among children taking HAART in Ethiopia was 363 (65.6%). Moreover, vitamin-D in-sufficiency was 199 (36%). A quarter of the children 140 (25.3%) were at grade-4 level liver toxicity while renal toxicity was 16 (2.9%). Level of anemia among children taking HAART in Ethiopia was also 275 (29.6%) (Figure-2).

### 4.3 Determinants of inflammation

Children who were on TDF+3 TC + EFV were 17.84 (95%CI = 16.98, 18.82) times risk of high level of CRP compared to those who were on ABC+3 TC + NVP. Patients who were not virally suppressed were at 2.2 (95%CI = 1.67, 2.88) times risk of high level of CRP compared to those whose were not suppressed. Children with SGP>46IU were at 1.20 (95%CI = 1.14, 1.93) times risk of high level of CRP compared to those with SGPT of <46 IU (Table-3).

**Table-3:** Determinants of inflammation among Children taking HAART in Ethiopia.

Variable		Vitamin-D				CRP			
		Sig.	AOR	95% C.I		Sig.	AOR	95% C.I	
				Lower	Upper			Lower	Upper
Gender	Female	0.89	1.02	0.69	1.52	0.07	0.64	0.39	1.03
	Male	Ref.							
Residency	Urban	0.81	0.92	0.48	1.74	0.38	0.68	0.29	1.59
	Rural	Ref.							
Education	No formal education	Ref.				0.26			
	Primary school education	0.92	0.96	0.39	2.35	0.17	0.47	0.15	1.39
	secondary/high school education	0.35	1.33	0.72	2.46	0.96	1.01	0.48	2.11
Family HIV status (Positive)	Yes	Ref.							
	No	0.68	0.79	0.26	2.38	0.06	0.55	0.29	1.04
Type of HAART	ABC,3 TC, NVP	Ref.							
	AZT,3 TC, EFV	0.11	6.85	0.62	74.69	0.04	0.16	0.02	0.94
	d4t,3 TC, NVP	0.53	1.42	0.45	4.44	0.07	0.36	0.12	1.11
	TDF,3 TC, EFV	0.02	4.10	1.64	6.89	0.00	17.84	16.98	18.82
HAART substitution	No	0.80	1.06	0.65	1.74	0.26	1.36	0.79	2.35
	Yes	Ref.							
Functional status	Ambulatory	Ref.							
	Bed Ridden	0.79	1.07	0.63	1.79	0.16	1.59	0.83	3.07
WHO clinical stage	I	Ref.							
	II	0.93	1.05	0.26	4.18	0.10	6.60	0.67	65.19
	III	0.76	0.81	0.20	3.14	0.17	4.76	0.49	46.23
	IV	0.91	1.08	0.24	4.83	0.21	4.96	0.39	61.89
Adherence	Good	Ref.							
	Fair	0.92	0.96	0.39	2.32	0.54	0.69	0.20	2.32
	Poor	0.73	0.85	0.33	2.16	0.79	0.84	0.23	3.09
Viral suppression	Not suppressed	1.00	3.2	0.00		0.00	2.22	1.67	2.88
	suppressed	Ref.							
Age	≤1	Ref.							
	2–6	1.00	7.75	0.00		1.00	4.71	0.00	
	7–11	0.16	0.67	0.38	1.18	0.79	1.09	0.55	2.14
	12–15	0.04	0.63	0.40	0.99	0.10	0.63	0.36	1.09
Duration on HAART (Month)	≤10	Ref.							
	11–40	1.00	1.45	0.00		0.13	96.70	0.25	36836.13
	41–70	0.99	0.00	0.00		0.28	15.63	0.10	2346.73
	71–100	0.99	0.00	0.00		0.41	8.01	0.05	1191.48
	101–131	0.99	0.00	0.00		0.35	10.92	0.07	1678.64
	>132	0.99	0.00	0.00		0			
Hgb (g/dl)	Anemic	0.74	1.06	0.72	1.56	0.01	1.21	1.15	1.96
	Normal	Ref.							
SGOT	≤200	0.66	1.20	0.51	2.83	0.99	0.00	0.00	
	>201	Ref.							
CD4 count	≤200.00	0.01	2.16	1.31	4.26	0.38	1.52	0.59	3.92
	>201.00	Ref.							
SGPT	>46.00	0.98	0.99	0.61	1.60	0.04	1.20	1.14	1.93
	≤45.00	Ref.							
Creatinine	>1.31	0.03	5.94	1.18	29.89	0.99	0.00	0.00	
	≤1.30	Ref.							
Urea	≤24.00	0.97	1.00	0.67	1.50	0.23	0.74	0.45	1.20
	>25.00	Ref.							

Key: AOR = Adjusted Odds Ratio; CI=Confidence Interval; HAART=Highly Active Antiretroviral Therapy; Hgb = Hemoglobin; CRP=C-Reactive Protein.

**Figure-3:**
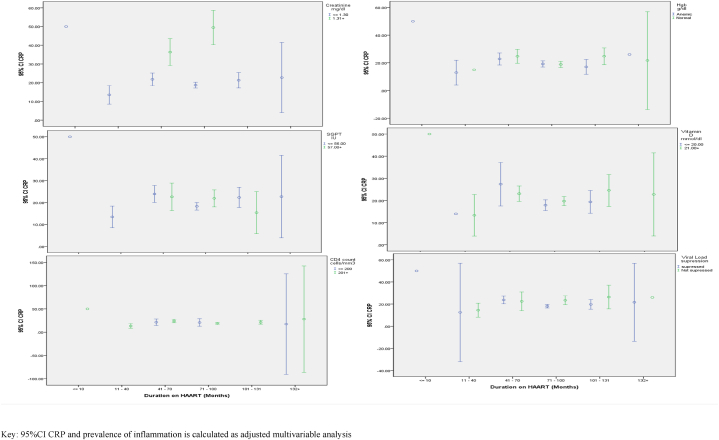
Determinants of Inflammation among children taking HAART in Ethiopia. Key: 95%CI CRP and prevalence of inflammation is calculated as adjusted multivariable analysis

As showed in Table-4, children taking TDF+3 TC + EFV were also at 4.10 (95%CI = 1.64, 6.89) times risk of vitamin-D deficiency compared to those who are taking ABC+3 TC + NVP. Children with CD4 count of <200 cells/mm^3^ were at 2.16 (95%CI = 1.31, 4.26) times risk of vitamin-D Deficiency compared to those with CD4 count of >200 cells/mm^3^. Moreover, patients with Creatinine level of >1.31 were at 5.94 (95%CI = 1.18, 29.89) times risk of vitamin-D deficiency compared to those with Creatinine level of <1.30 (Table-3 and Figure-3).

**Table-4:** Determinants of Renal and liver toxicity among children taking HAART in Ethiopia.

Variable		Liver toxicity				Renal Toxicity			
Variable		AOR	Sig.	95% CI		AOR	Sig.	95% CI	
				Lower	Upper			Lower	Upper
Gender	Female	0.85	0.35	0.16	0.46	0.01	0.89	3.36	3.87
	Male	Ref.							
Residency	Urban	0.15	0.69	0.41	0.63	0.52	0.47	3.27	7.10
	Rural	Ref.							
Education	No formal education	0.01	0.89	0.70	0.80	0.50	0.47	15.18	32.55
	Primary school education	0.24	0.61	0.62	0.37	1.76	0.18	2.42	12.61
	secondary/high school education	Ref.							
Family HIV status	Yes	0.01	0.90	0.43	0.38	4.07	0.04	2.30	6.09
	No	Ref.							
	Don't Know	Ref.							
Type of HAART	ABC,3 TC,EFV	0.43	0.50	1.99	4.03	1.21	0.27	84.22	23.64
	BC,3 TC,LPV/r			2.85	6.25	0.05	0.81	260.16	205.39
	ABC,3 TC,NVP	0.53	0.46	17.50	17.50	0.07	0.78	266.48	201.30
	AZT,3 TC,EFV	0.83	0.36	1.48	4.06	17.63	0.00	18.25	27.54
	AZT,3 TC,LPV/r	0.92	0.33	1.90	5.58	0.22	0.63	135.23	82.17
	AZT,3 TC,NVP	0.79	0.37	1.50	4.01	22.48	0.00	13.93	29.31
	d4t,3 TC,EFV	3.30	0.06	0.20	5.48	4.34	0.03	2.51	6.80
	d4t,3 TC,NVP	3.00	0.08	0.31	5.18	18.91	0.00	4.87	27.74
	TDF,3 TC,EFV	0.86	0.35	1.47	4.13			19.62	19.62
	TDF,3 TC,NVP	Ref.							
HAART substitution	Yes	4.66	0.03	1.84	6.04	0.59	0.44	3.67	8.43
	No	Ref.							
Adherence	Good	0.06	0.80	0.60	0.78	2.31	0.12	17.36	2.18
	Fair	0.09	0.75	0.86	0.62	2.07	0.15	16.38	2.50
	Poor	Ref.							
Viral suppression	suppressed	0.01	0.91	0.44	0.39	1.26	0.26	1.95	7.19
	Not suppressed	Ref.							
Functional status	Bedridden	3.56	0.05	2.01	4.71	0.39	0.53	2.59	5.02
	Ambulatory	Ref.							
Age	≤1	1.13	0.28	5.56	1.65	0.00	0.96	227.31	239.31
	2–6	5.55	0.01	1.01	0.09	1.86	0.17	1.20	6.72
	7–11	1.64	0.19	0.59	0.12	0.09	0.75	4.28	5.91
	12–16	Ref.							
Duration on HAART	≤10	1.58	0.20	7.10	1.54	0.00	0.97	246.70	254.75
	11–40	0.55	0.45	4.26	1.92	0.00	0.99	112.25	112.85
	41–70	4.09	0.04	5.00	0.08	0.02	0.88	100.31	86.77
	71–100	3.03	0.08	4.62	0.27	0.01	0.90	99.20	87.65
	101–131	3.29	0.06	4.78	0.18	0.03	0.84	104.00	85.12
	>132	Ref.							
Hgb	Anemic	0.29	0.58	0.40	0.22	0.11	0.73	2.31	3.28
	Normal	Ref.							
CD4 count	≤200	0.02	0.87	0.55	0.65	0.29	0.58	4.40	7.75
	>200	Ref.							
CRP	No-Inflammation	0.02	0.87	0.43	0.37	0.67	0.41	14.11	5.77
	Inflammation	Ref.

### 4.4 Determinants of liver and renal toxicity among children taking HAART in Ethiopia

Determinants of liver toxicity were history of HAART substitution 4.66 (95%CI: 1.84, 6.04) and being bedridden 3.56 (95%CI = 2.01, 4.71). Renal toxicity was associated with family HIV status as being positive 4.07 (95%CI = 2.30, 6.09). Children on AZT+3 TC + EFV, AZT+3 TC + NVP, d4t+3 TC + EFV and d4t+3 TC + NVP were at 17.63 (95%CI = 18.25, 27.54), 22.48 (95%CI = 13.93, 29.31), 4.34 (95%CI = 2.51, 6.80), 18.91(95%CI = 4.87, 27.74) times risk of renal toxicity compared to those who are taking TDF+3 TC + NVP (Table-4).

### 4.5 Determinants of hematologic toxicity among children taking HAART in Ethiopia

As showed in Table-5, Factors associated with hemoglobin was gender 0.66 (95%CI = 0.46, 0.95) and type of HAART; AZT+3 TC + EFV 4.92 (95%CI = 1.86, 12.70) compared to those taking TDF+3 TC + EFZ. Children living in rural were at 3.53 (95%CI95%CI = 1.25, 9.92) times risk of low CD4 count. Moreover, children who were at WHO clinical stage II, III and IV were at 12.94 (95%CI95%CI = 2.47, 16.46), 11.26 (95%CI = 1.49, 85.90), and 57.6 (95%CI = 4.2,79.50) times risk of low CD4 count compared to clinical stage I. Children who were on HAART in the range from 101 to 131 and > 132 month were at risk of low CD4 count which accounted 10.80 (95%CI = 3.80, 14.36) and 8.70 (95%CI = 4.91, 18.65) respectively.

**Table-5:** Determinants of Hematologic toxicities among children taking HAART in Ethiopia.

Variable		Hgb				CD4			
		Sig.	AOR	95% C.I		Sig.	AOR	95% C.I	
				Lower	Upper			Lower	Upper
Gender	Female	0.02	0.66	0.46	0.95	0.44	1.31	0.65	1.97
	Male	Ref.							
Residency	Rural	0.34	1.32	0.73	2.39	0.01	3.53	1.25	5.81
	Urban	Ref.							
Education status	No formal education	Ref.							
	Primary school education	0.16	0.56	0.24	1.27	0.99	3.33	0.00	6.66
	secondary/high school education	0.60	0.86	0.49	1.51	0.22	1.78	0.69	2.87
Family status	Yes	0.35				0.84			
	No	0.19	1.34	0.85	2.11	0.55	1.28	0.56	2
	Don't Know	Ref.							
HAART type	ABC,3TCNVP	0.11				0.50			
	AZT,3 TC, EFV	0.04	4.92	1.86	12.70	0.28	0.34	0.04	0.64
	d4t,3 TC, NVP	0.27	1.52	0.71	3.21	0.65	1.30	0.41	2.19
	TDF,3 TC, EFZ	Ref.							
Substitution	No	0.70	1.09	0.69	1.71	0.83	1.09	0.46	1.72
	Yes	Ref.							
Functional status	Ambulatory	Ref.				0.44			
	Bedridden	0.25	0.75	0.47	1.21	0.91	1.06	0.37	1.75
WHO clinical stage	I	Ref.							
	II	0.62	1.38	0.37	5.15	0.00	12.94	2.47	23.41
	III	0.58	1.43	0.38	5.29	0.01	11.26	1.47	21.05
	IV	0.94	1.05	0.25	4.27	0.00	57.68	4.20	111.16
Adherence	Good	Ref.				0.99			
	Fair	0.85	1.07	0.48	2.36	0.92	1.07	0.24	1.9
	Poor	0.85	1.08	0.46	2.49	0.97	1.03	0.21	1.85
Viral suppression	suppressed	0.53	0.85	0.53	1.38	0.00	3.54	1.47	5.61
	Not suppressed	Ref.							
Age Duration on HAART	≤1	0.46				0.98			
	2–6	1.00	4.26	0.00		1.00	5.88	0.00	11.76
	7–11	0.11	1.52	0.90	2.54	0.88	1.07	0.39	1.75
	12–15	Ref.							
	≤10	0.36				0.00			
	11–40	1.00	0.00	0.00		1.00	7.90	0.00	15.8
	41–70	0.19	0.11	0.00	3.13	0.99	1.32	0.00	2.64
	71–100	0.28	0.27	0.02	2.95	0.04	10.80	3.80	17.8
	101–131	0.43	0.38	0.03	4.09	0.00	8.70	4.91	12.49
	>132	Ref.							

## 5 Discussion

Immune activation has been demonstrated to be a significant contributor to HIV disease progression in multiple studies [18,21,22]. It was observed that this immune activation was associated with increased levels of viral load [23]. Previous reports revealed, CRP being an acute phase reactant will increase in patients with faster HIV disease progression [24,25]. Also it was reported that, one month after stopping treatment, HIV RNA levels were correlated with increases in CRP levels and were subsequently associated with an increased risk of all-cause mortality [21,26]. In our current study, HAART induced inflammation among HIV positive children in Ethiopia were found to be 363 (65.6%). This was consistent with a study conducted in Uganda that concluded a high level of inflammation (62.21%) among people taking HAART and a significant association of immune activation as measured by hsCRP levels with HIV disease progression [25]. In the current study, patients who were taking TDF+3 TC + EFV were at 17.84 times risk of inflammation compared to those who are taking ABC+3 TC + NVP. This was not consistent with other previous studies which revealed TDF and ABC to be safe drugs and 3 TC is a common drug for the entire regimen. The difference in this context was EFV and NVP while previous reports revealed NVP as more toxic drug compared to EFV [4,27]. This might be explained by the effect of combined therapy which could exacerbate the level of inflammation. Moreover, the previous studies were conducted among adult population which might have difference due to the physiological factors among children. In the current study, patients who were not virally suppressed were at 2.2 times risk of inflammation compared to those whose were not suppressed which was consistent with previous study conducted in Uganda [18]. Patients with liver toxicity were at 1.20 times risk of inflammation [28]. This could be explained by the fact that the different levels of toxicities could increase inflammation [21,29,30].

Vitamin-D is a pro-hormone that has anti-inflammatory effects. Vitamin-D deficiency (VDD) is associated with greater inflammation by up-regulation of inflammatory markers like, IL-6, TNF-α, activated monocyte phenotypes (CX3CR1+and CCR2+) in HIV-infected patients, which have been related to tissue dysfunction, comorbidity development, AIDS progression, and death in HIV-infected individuals [22]. Our current study showed, Vitamin-D insufficiency among children taking HAART in Ethiopia was 199 (36%). This was consistent with a study conducted in Tanzania which showed 27.1% [22]. Studies reported, HAART especially efavirenz may impair vitamin D metabolic pathways. Our study also showed children taking TDF+3 TC + EFV were at 4.10 times risk of vitamin-D deficiency compared to those who are taking ABC, 3 TC, NVP. Previous studies in Zimbabwe also revealed low CD4 count (<200/μl) and current efavirenz use were independently associated with severe VDD [19,31]. This was consistent with our current study which revealed, Children with CD4 count of <200 cells/mm^3^ were at 2.16 times risk of vitamin-D Deficiency compared to those with CD4 count of >200 cells/mm^3^. Moreover, in our current study, children with renal failure were at 5.94 times risk of vitamin-D deficiency [32]. Despite, screening and providing vitamin-D supplementation for children are not routinely done in HIV infected children in low-income settings though this is recommendation by the Endocrine Society [33].

In our study, a quarter of the study participants 140 (25.3%) were at grade-4 level liver toxicity Another cross sectional study conducted in Ethiopia revealed prevalence of liver enzyme abnormality was 20.1% among HAART experienced and HAART patients [34]. This was also consistent with a study conducted in Ethiopia, Debre Birhan Referral Hospital which showed 18.2% of liver toxicity [9]. Determinants of liver toxicity were history of HAART substitution and being bedridden with a risk of 4.66 and 3.56 times. This could also be explained by one of the potential reasons for substitution of HAART could be due to toxicity. Being bedridden could also be explained by the potential impact of toxicity leading to the high rate of morbidity among children taking HAART.

In the current study, renal toxicity was 16 (2.9%). This was lower compared to a study conducted in Gonder University Hospital, Ethiopia which revealed 11.7% [35]. This might be due to the fact that, the study conducted among adult population and with a different regimen. Children taking AZT+3 TC + EFV, AZT+3 TC + NVP, d4t+3 TC + EFV and d4t+3 TC + NVP were at 17.63; 22.48, 4.34 and 18.91times risk of toxicity compared to those who are taking TDF+3 TC + NVP. This implies, almost all the regimen have some level of renal toxicity which could be linked to the combination effect of the drugs [36]. In a meta-analysis of 17 studies examining TDF safety, a significantly greater loss of kidney function among the TDF recipients, compared with control subjects [37]. This was also consistent with our study.

Anemia among HIV infected children is an emerging public health problem. In our current study, anemia among children taking HAART in Ethiopia was 275 (29.6%). However, the study conducted in Tigray region, Ethiopia revealed a lower 17% prevalence of anemia among children taking HAART. This result is higher than the findings reported in Northwest Ethiopia (2.3%) [38], Southwest Ethiopia (14.3%) [39], Southern Ethiopia (6.5%) [40], Uganda (4.8%) [41] and India (8%) [42]. This variation could be attributed to the difference in socioeconomic status, geographical factors (altitude), nutritional factors, seasonality, WHO clinical staging of HIV disease, time of the study, or a combination of these factors, which are known to affect anemia. Factors associated with anemia were being female 0.66. This might be explained by the inherent differences among gender. In our current study, children taking AZT+3 TC + EFV were at 4.92 times risk of acquiring anemia compared to those taking TDF+3 TC + EFZ [43]. This was consistent with other studies conducted in Tigray region, Ethiopia and also a study conducted in Black Lion Hospital, Ethiopia. Given the high mortality rate of severely anemic children living with HIV/AIDS on HAART, this particular finding is a grave concern.

### 5.1 Limitation of the study

This study was conducted in 43 systematically selected health facilities and hence one of the strengths is its generalizability. Children in orphanages, those who are attending health facilities and special schools were not part of this study. The limited information on potential HAART substitution might introduce information bias. The different laboratory biomarker testing methods to evaluate toxicity and inflammation among children had their limitations on specificity and sensitivities among the different testing platforms. Hence, the interpretation of the finding has to consider this limitation in to account.

## 6 Conclusion

The high level of inflammation and liver toxicity calls for the program to consider safer drugs for pediatric patients. Moreover, the high level of vitamin-D deficiency requires program level supplement. The impact of TDF+3 TC + EFV on inflammation and vitamin-D deficiency calls for the program to revise these regimens.

## Author contribution statement

Yimam Getaneh Misganie, Kuntaman Kuntaman: Conceived and designed the experiments; Analyzed and interpreted the data; Wrote the paper.

Tadesse Lijessa, Tigist Getahun: Performed the experiments; Contributed reagents, materials, analysis tools or data; Wrote the paper.

Siti qamariyah khairunisa, Dominicus Husada: Performed the experiments; Analyzed and interpreted the data; Wrote the paper.

Maria Inge Lusida: Wrote the paper; Conceived and designed the experiments; Contributed reagents, materials, analysis tools or data.

## Additional information

No additional information is available for this paper.

## Availability of data and materials

Data will be available upon reasonable request of the corresponding author of the study.

## Competing interests

The authors declare that they have no competing interests.

## Author Contributions

All authors made a significant contribution to the work reported, whether that is in the conception, study design, execution, acquisition of data, analysis and interpretation, or in all these areas; took part in drafting, revising, or critically reviewing the article; gave final approval of the version to be published; have agreed on the journal to which the article has been submitted; and agree to be accountable for all aspects of the work.
